# Evaluation of intestinal parasites as a causative agent of diarrhea in children and some treatments used in Babylon Governorate

**DOI:** 10.11604/pamj.2023.46.29.40536

**Published:** 2023-09-19

**Authors:** Khulood Abdul-Majeed Mohammed Jafeer, Saba Fadhil Ali Malaa, Zainab Abed Mohsen Al-Aboobi, Naiel Abbass Kadhiem Alkhafaji

**Affiliations:** 1 Community Health Department, Technical Institute of Babylon, Al Furat Al-Awsat Technical University, Babylon, Iraq,; 2 Community Health Department, Technical Institute of Karbala, Al Furat Al-Awsat Technical University, Karbala, Iraq

**Keywords:** Parasitic diarrhea, flagyl, pyrantel, albendazole

## To the editors of the Pan African Medical Journal

Given that children with malnourished or weakened immune systems are the age group most susceptible to this ailment, diarrhea is therefore regarded as the second-leading cause of death for children worldwide. However, a large proportion can be prevented through the provision of safe drinking water and adequate and clean sanitation facilities [1]. Over the years, many drugs to treat parasitic infections in animals and humans have been developed. With the development of parasite resistance to antiparasitic drugs, alternative treatment options for parasitic diseases are being increasingly researched. Giardiasis and amebiasis are major diarrheal diseases that spread all over the world. Flagyl (metronidazole and tinidazole) has been the most widely used drug in recent years [2,3]. Pyrantel is a drug used to treat parasitic worms, including ascariasis and pinworms. So increasing levels of resistance to albendazole and mebendazole are of concern [4,5]. No systematic review has assessed the effectiveness of combining 5-nitroimidazoles or albendazole with other drugs [6,7]. Therefore, the idea of the research was to conduct an epidemiological survey of the parasitic infection with some variables and assess the therapeutic effect of some of the drugs that were taken in the study.

During the study, samples of stool were collected from children who suffered from diarrhea and sent to the laboratory for analysis to diagnose the pathogen. The study was conducted over a span of one year. The highest percentage was 32% infected with *G. lamblia*, 28% infected with *E. histolytica*, pinworm infection reached 23%, and *A. lumbricoides* infection rate reached 11.3%, followed by 6% infected with *Cryptosporidium parvum*. This difference occurs when taking the sample into account and the type of study. Some researchers mention that the difference in space and time played an important role because of the different rates of infection with some endemic parasites in the study areas. The results demonstrate that there was no significant difference between males and females according to the type of parasitic infection, and were similar to [6,7]. Hormonal changes played a role in the difference in infection rates. Also, the results differed from [8,9].

It was revealed that the age group of 6-10 years had the highest infection rate 46.6%, while the lowest infection age was five years and reached 14%. We determine that there is a significant link between family income and parasitic diarrhea, in which the participants who have a middle income are at higher risk for parasitic diarrhea with 2.154 times more than those who have a high income at a P-value of 0.002. The participants who have a household size of 6 to 10 persons and >11 individuals are at higher risk for parasitic diarrhea, with 3.055 and 3.150 times, respectively, more than those who have a household size of three to five individuals. As for other variables, the present results reveal that there is no significant association with parasitic diarrhea (P-value >0.05). Although there is a difference in infection rates, according to the difference in housing between the countryside and the city, most of the samples were city residents, at a rate of 54%, and most of them depended on drinking liquefied water, at a rate of 58%. Similar research found that the incidence of giardiasis is the highest, followed by *E. histolytica* and *A. lumbricoides*, especially among mothers with limited income and little education. The social and economic status of the children's parents was studied, as well as the difference in the geographical area, taking into account the presence of double infections, and this was supported by each of them [8-10].

By examining stool samples before and after treatment, the effect of certain drugs on parasites was determined, as shown in Table 1. There was a significant effect of the metronidazole drug on *G. lamblia* by 90%, followed by the *E. histolytic* by 81%, then *A. lumbricoides* by 76%, and pinworms by 6%, while the effect of flagyl was 17% on the *C. parvum*. Pyrantel was ineffective against *C. parvum*, but showed a significant effect against *E. vermicularis* (74%), *A. lumbricoides* (65%), and *G. lamblia* (38%). The study also showed the effect of albendazole with high efficiency on most of the study parasites and the percentages were as follows: 91% *Enterobius vermicularis*, 88% *A. lumbricoides*, 67% *C. parvum*, 21% *G. lamblia*, and 12% *E. histolytica*. Based on the drug's action on several parasitic infection-related conditions such as lactose intolerance, tinidazole, and irritable bowel syndrome, the results corroborated [9, 10], which demonstrated the immediate effect of albendazole and metronidazole on giardiasis. Furazolidone, quinacrine, or albendazole had rarely been used because of their potential toxicity and decreased efficacy.

**Table 1:** results of the effect of treatment before and after infection and the type of parasite in the Babylon Province, Iraq, during the period of March 2021 to August 2022

Drug	Classification of parasite	Number of infected patients		Percentage of effective treatment
		Before treatment	After treatment	
Metronidazole	*Entameabia histolytica*	42	8	81%
	*Giardia lamblia*	48	5	90%
	*Ascaris lumbricoides*	17	4	76%
	*Enterobius vermicularis*	35	33	6%
	*Cryptosporidium parvum*	6	5	17%
Pyrantel	*Entameabia histolytica*	42	38	10%
	*Ascaris lumbricoides*	17	6	65%
	*Giardia lamblia*	48	30	38%
	*Enterobius vermicularis*	35	9	74%
	*Cryptosporidium parvum*	6	6	0%
Albendazol	*Ascaris lumbricoides*	17	2	88%
	*Enterobius vermicularis*	35	3	91%
	*Giardia lamblia*	48	38	21%
	*Entameabia histolytica*	42	37	12%
	*Cryptosporidium parvum*	6	2	67%

**Conclusion:** the study analyzed 266 fecal samples from children in Babylon Province and identified six intestinal parasites, with *G. lamblia* and *E. histolytica* being the most prevalent. The cross-sectional study found that urban inhabitants were more likely to suffer from parasitic diarrhea. The treatments used to address the issue were metronidazole and albendazole, which were found to be highly effective in treating most of the parasites.
